# Validation of the Chinese Version of the Procrastination at Work Scale

**DOI:** 10.3389/fpsyg.2021.726595

**Published:** 2021-09-16

**Authors:** Jiayan Wang, Chaoping Li, Xue Meng, Doudou Liu

**Affiliations:** Institute of Organization and Human Resources, School of Public Administration and Policy, Renmin University of China, Beijing, China

**Keywords:** procrastination at work, scale validation, measurement invariance, Chinese adults, work engagement, task performance

## Abstract

The purpose of this study was to validate the Chinese version of the Procrastination at Work Scale (PAWS), a recently developed scale aimed at assessing procrastination in the work context. We translated the PAWS into Chinese and conducted exploratory factor analysis on participants in sample A (*N* = 236), resulting in a two-factor solution consistent with the original PAWS. In sample B (*N* = 227), confirmatory factor analysis showed that a two-factor, bifactor model fit the data best. Configural, metric, and scalar invariance models were tested, which demonstrated that the Chinese version of the PAWS did not differ across groups by gender, age, education, or job position. Validity testing demonstrated that the scale relates to work engagement, counterproductive work behavior, task performance, workplace well-being, and organizational commitment. This study indicated that the Chinese version of the PAWS could be used in future research to measure procrastination at work in China.

## Introduction

Procrastination is defined as the voluntary delay of an intended course of action despite expecting negative consequences for the delay (Steel, 2007; Klingsieck, 2013a). Procrastinators tend to demonstrate larger intention-action gaps compared to non-procrastinators (Steel et al., 2001). Procrastination is often thought of as a self-regulatory failure or a behavior driven by emotion (Sirois and Pychyl, 2013). Researchers have found that procrastination, a common behavior, exists in academic, life, and work domains (Klingsieck, 2013b). More than 70% of students engage in procrastination (Schouwenburg, 1995), and more than 20% of university students thought procrastination to be a very serious problem with negative effects on their learning engagement and academic performance (Kim and Seo, 2015; Metin et al., 2019).

A large amount of literature exists on procrastination among students (Schouwenburg, 1995; Steel, 2007; Sirois and Tosti, 2012; Klingsieck, 2013a; Kim and Seo, 2015; van Eerde, 2016), but few research has been done on procrastination at work (Metin et al., 2016, 2019). However, procrastination is embedded in many aspects of organizational life. Procrastination at work is defined as “delay of work-related action by engaging (behaviorally or cognitively) in non-work-related actions, with no intention of harming the employer, employee, workplace, or client” (Metin et al., 2016). Studies have reported that employees spend on average 1.5–3 h per day on personal activities or Internet surfing during their working time (Sharma and Gupta, 2003; Paulsen, 2013), which may cause a yearly loss of $8,875 per employee (D'Abate and Eddy, 2007). An H&R Block survey showed that procrastinating on taxes costs people on average $400 per year because of rushing and consequent errors, resulting in over $473 million in overpayments in 2002 (Steel, 2007). As a prevalent behavior at work, procrastination is influenced by task characteristics and personality; procrastination can also affect mood and performance (Lay, 1992; Lay and Brokenshire, 1997; Steel, 2007). Moreover, high level stress and boredom are associated with increased procrastination at work and are also related to decreased work engagement (Metin et al., 2019). Over 95% of procrastinators wish to get rid of this harmful behavior at work (O'Brien, 2002). Considering the high costs and negative impacts of procrastination, understanding this behavior will help us to counteract it in work environments.

One limit to research on workplace procrastination is the lack of a specific, reliable, and valid measurement tool (Metin et al., 2016), which makes it difficult to measure procrastination in the workplace accurately. In the literature on procrastination at work, most previous studies used the general or academic procrastination scale to measure procrastination in the work context. For example, Gupta et al. (2012) used the general procrastination scale to measure workplace procrastination. To address this lack, Metin et al. (2016) developed a Procrastination at Work Scale (PAWS) to assess procrastination in the work domain. The PAWS consists of two related dimensions, soldiering, and cyberslacking. Soldiering (eight items) is any kind of offline off-task activity such as taking long coffee breaks, gossiping, or daydreaming (Metin et al., 2016), and is defined as “avoidance from work tasks for more than 1 h a day without aiming to harm others or shifting work onto colleagues” (Paulsen, 2013). Cyberslacking (four items) is online off-task activity, such as reading blogs for personal interest (Metin et al., 2016); cyberslacking emerged with the wide use of technology at work (Vitak et al., 2011). Cyberslacking is harder to measure than soldiering, because cyberslacking can look like work—employees just need to sit in front of a computer and click a mouse.

The two-factor PAWS has been translated and validated in Dutch, Slovenian, Croatian, Czech, Turkish, Finnish, English, and Ukrainian (Metin et al., 2016, 2019). Measurement invariance was tested across culture groups in previous studies, however, was not tested across groups by gender, age, education, and job position. Measurement invariance tests across groups by gender, age, education, and job position are necessary because they increase the accuracy of measuring procrastination at work and the comparability across groups.

In the context of Chinese culture, the reliability and validity of the two-factor PAWS has been untested until now. When using a scale, the influences of culture should not be ignored, because a scale may be able to measure dynamics for only a specific cultural background. For that reason, we found it necessary to estimate factor structure among Chinese employees. The current study comprised two stages: translating the PAWS into Chinese and testing its reliability, construct, and nomological validity among Chinese employees, and testing measurement invariance across multiple groups. In stage 1, we translated the PAWS using the classic back-translation method, and then conducted exploratory factor analysis (EFA) to assess the scale structure. The results of the EFAs supported two distinct, significantly correlated factors. In stage 2, we conducted confirmatory factor analyses (CFA) to assess factor structure and tested its validity through assessing the relationships between the PAWS and related constructs. Finally, we tested measurement invariance across different groups.

## Materials and Methods

### Stage 1: Validating the Chinese Version of the Procrastination at Work Scale

#### Scale Translation

The English version of the PAWS was translated into Chinese following the classic back-translation method (Brislin, 1980). First, all items were translated into Mandarin by two bilingual authors, and we compared the Mandarin items and reached a consensus on the translation of the scale. Second, the Mandarin items were back-translated by another bilingual author, after which all four authors compared the back-translated versions with the original English items to detect any discrepancies and inconsistencies. Finally, we discussed the inconsistencies and made minor revisions until we reached a consensus.

The measurement of procrastination at work used in stage 1 was the translated Chinese version of the PAWS (PAWS-C). Respondents were asked to indicate their level of work procrastination on a five-point Likert-type scale, in which 1 = *never*, 2 = *seldom*, 3 = *sometimes*, 4 = *often*, and 5 = *always*.

#### Participants and Procedures

Snowball sampling was used to collect data. We created an online survey link and distributed it to 50 adults from various organizations enrolled in a part-time Master of Public Administration program at a prestigious University in North China, asking them to forward the link to their colleagues or employed friends who might be interested in participating. Participants were assured of anonymity and confidentiality. We administered 484 employee questionnaires comprising an information page, the 12-item Chinese version of the PAWS, the scales of related constructs, and a demographic questionnaire to determine age, gender, full-time work experience, occupation, average work hours per week, and job position. Based on the return of 484 questionnaires, 463 were valid, the valid rate was 95.66% (463/484).

Participants in this study were 463 working adults in different industries in China. All participants were required to be 18 years of age or older and to be currently employed. Participants were randomly split into two subsamples using the random split function in SPSS V26. Sample A (*N* = 236) was used in stage 1 for conducting EFA, and Sample B (*N* = 227) was used in stage 2 to test factor structure, validity, and measurement invariance.

Sample A was 55.93% male, and participants' average age was 34.29 years (*SD* = 8.22; range = 19–59 years). Only one participant (0.42%) had less than college education; 63.56% had a college degree (*n* = 150), and 36.02% had a Master's degree or Ph.D. (*n* = 85). Respondents' average full-time work experience was 11.97 years, and they worked 47.85 h per week on average. Most of them were first-line, middle, and senior managers (*n* = 142, 60.17%); others were front-line employees (*n* = 94, 39.83%). Sample B was 55.07% male with an average age of 34.68 years (SD =7.77; range = 22–55 years). Again, only one respondent (0.44%) had less than college education, while 63.00% had a college degree (*n* = 143) and 36.56% had a Master's degree or PhD (*n* = 83). Respondents' average full-time work experience was 11.91 years, and they worked on average 48.09 h per week. Most of them were first-line, middle, and senior managers (*n* = 136, 59.91%), while others were front-line employees (*n* = 91, 40.09%).

We compared samples A and B to test for between-group difference. A series of tests showed that the samples were not significantly different with regard to gender, age, education, full-time work experience, working hours per week, occupations, or job position (all *p* values > 0.05; gender, [${\text{χ}}_{\left(1\right)}^{2}$ = 0.04, *p* = 0.85]; age, [*t*_(461)_ = 0.53, *p* = 0.60]; education, [${\text{χ}}_{\left(4\right)}^{2}$ = 0.50, *p* = 0.97]; full-time work experience, [*t*_(461)_ = −0.07, *p* = 0.94]; working hours per week, [*t*_(458)_ = 0.21, *p* = 0.84]; occupations, [${\text{χ}}_{\left(5\right)}^{2}$ = 3.90, *p* = 0.56]; job position, [${\text{χ}}_{\left(5\right)}^{2}$ = 7.07, *p* = 0.22].

#### Analysis

To examine the dimensionality of the Chinese version of the PAWS, we conducted an EFA (principal component analysis). Consistent with Metin et al. (2016) and Metin et al., 2019) original studies, we expected the factors would be correlated to form an overall Procrastination at Work Scale, so we selected an oblique rotation (direct rotation; Fabrigar et al., 1999) in IBM SPSS V26 (Costello and Osborne, 2005).

### Stage 2: Confirmatory Factor Analysis and Validity of the PAWS

Our aims in stage 2 were to examine the factor structure of the PAWS using sample B with a series of CFAs, including a correlated two-factor model, a single factor model, a second-order two-factor model, and a bifactor model. To further test the validity of our measure, we calculated the Average Variance Extracted (AVE) values for the PAWS (Fornell and Larcker, 1981; Toro-Arias et al., 2021) and compared the square root of the AVE score with its correlations with other constructs. We also checked expected associations with occupational/career concepts (work engagement, counterproductive work behavior, task performance, workplace well-being, and organizational commitment), consistent with the original scale development study (Metin et al., 2016, 2019). During this stage, we also tested the measurement invariance of the PAWS using multigroup CFA across gender, age, education, and job position.

#### Participants

Sample B (*N* = 227) was used for the CFA and invariance testing and to provide initial evidence of validity by testing expected associations with theoretically similar constructs (work engagement and counterproductive work behavior) and constructs drawn from the nomological net (task performance, workplace well-being and organizational commitment).

#### Measures

The PAWS-C was included in an online questionnaire with demographic questions (identical to stage 1) and measures for assessing validity. Unless otherwise noted, items were rated on a five-point scale ranging from 1 = *strongly disagree* to 5 = *strongly agree*.

##### Procrastination at Work

We measured procrastination at work using the 12-item scale validated in stage 1. Cronbach's alpha was 0.91 in sample B. Cronbach's alpha were 0.90 for Soldiering and 0.86 for Cyberslacking. Each item was rated on a five-point Likert-type scale, ranging from 1 = *never* to 5 = *always*.

##### Work Engagement

We assessed work engagement using Schaufeli et al.'s (2019) three-item work engagement scale (UWES-3), comprising the following items: (1) “At my work, I feel bursting with energy” (vigor); (2) “I am enthusiastic about my job” (dedication); (3) “I am immersed in my work” (absorption). Schaufeli et al. (2019) provided good reliability estimates, higher than 0.70 (Schaufeli et al., 2019). Metin et al. (2018) provided reliability estimates higher than 0.80 significantly associated with procrastination at work. Good reliability was also demonstrated for the Chinese version of this scale (Meng et al., 2020). In our study, total score reliability was 0.88.

##### Counterproductive Work Behavior

Counterproductive work behavior was assessed with eight items (for example, “Spent time on tasks unrelated to work”) from the study by Dalal et al. (2009). Counterproductive work behavior was significantly associated with procrastination at work (Metin et al., 2016). Good reliability, higher than 0.80, was demonstrated for the Chinese version of this scale (Bai et al., 2016). In our study, reliability was 0.83.

##### Task Performance

Task performance was assessed with four items (for example, “I always complete job assignments on time”) from the study by Gong et al. (2009). Task performance was reported to be significantly associated with procrastination at work (Metin et al., 2019). Gong et al. (2009) reported reliability estimates higher than 0.90 for the Chinese version of the scale. In our study, reliability was 0.80.

##### Workplace Well-Being

Workplace well-being was assessed with six items (for example, “I find real enjoyment in my work” and “In general, I feel fairly satisfied with my present job”) from the study by Zheng et al. (2015). Procrastination at work as avoidance behavior is linked to diminished well-being and lower performance (Sirois and Tosti, 2012; Eerde and Venus, 2018). Zheng et al. (2015) reported reliability estimates higher than 0.80 for the Chinese version of the scale. In our study, reliability was 0.88.

##### Organizational Commitment

Organizational commitment was assessed with six items (for example, “I do feel a strong sense of belonging to my organization” and “This organization has a great deal of personal meaning for me”) from Allen and Meyer (1996). Allen and Meyer (1996) provided reliability estimates higher than 0.73. Li et al. (2020) also reported reliability estimates higher than 0.80 for the Chinese version of the scale. In our study, reliability was 0.84.

#### Analysis

We used CFA with the lavaan package in R3.6.3 (Rosseel, 2012; R Core Team, 2015) to evaluate the factor structure of the PAWS using maximum likelihood estimations to examine the goodness of fit of the Chinese version. This analysis was run to test a series of models in order to assess the most appropriate factor structure of the PAWS. We used fit indices χ^2^ (with a significant *p* value), χ^2^/^df^ ratio, comparative fit index (CFI), Tucker-Lewis index (TLI), root mean square error of approximation (RMSEA), and standardized root mean square residual (SRMR) to evaluate the goodness of fit of the models. Criteria for the χ^2^, χ^2/df^, CFI, TLI, and RMSEA have ranged from χ^2^ (*p* < 0.05), χ^2^/df ≤ 3, CFI ≥ 0.90, TLI ≥ 0.90, RMSEA and SRMR < 0.80 (Hu and Bentler, 1999; Kline, 2005). We used Akaike's Information Criterion (AIC) to compare different possible models and determine which one was the best fit for the data, with a lower AIC indicating a better fit (Raferty, 1995; Kline, 2005; Vrieze, 2012). We also use the Bifactor Indices Calculator (Dueber, 2017) computed various statistical indices relevant to evaluating bifactor models including ECV, Omega (Ω), OmegaH (ωH), OmegaHS (ωHS), IECV and PUC to strength the results.

Multigroup CFA tested measurement invariance across participants from various groups. We created dichotomous categorical variables for conducting invariance tests for gender, age, education, and job position (Milfont and Fischer, 2010; Schoot et al., 2012). Similar to previous studies (Duffy et al., 2019; Xu and Li, 2021), our gender comparison was between men and women, and for age we used two groups: low (those whose response was ≤ mean age, 34.68) and high (those whose response was > mean age, 34.68). We split education level into two groups, undergraduate degree and below and master's degree or above; for job position, we compared front-line employees and managers. We tested the invariance of several group classifications for equality of the overall factor structure (configural invariance), equality of item factor loadings (metric invariance) and equality of item intercepts (scalar invariance; Vandenberg and Lance, 2000).

For convergent validity, the focal construct should be empirically relayed to theoretically linked constructs such that it retains its uniqueness but reflects the underlying similarities with those related constructs (Campbell and Fiske, 1959). The PAWS is theoretically related to work engagement and counterproductive work behavior (Metin et al., 2016, 2019); therefore, we expected measures of work engagement and counterproductive work behavior to correlate significantly with the PAWS. We also checked that the AVE values for the PAWS were higher than 0.50. An AVE lower than 0.50 means the items explain more errors than the variance in the constructs. For any measurement model, an AVE must be calculated for each construct and must be at least 0.50. For discriminant validity, we checked whether the square root of the AVE for the whole PAWS was higher than the correlation between the PAWS and related constructs drawn from the nomological net, such as task performance, workplace well-being, and organizational commitment. The same constructs and their expected strong correlation with the PAWS were used to confirm the nomological validity of the Chinese version of the PAWS.

## Results

### Exploratory Factor Analysis (Sample A)

The Kaiser-Meyer-Olkin measure of sample adequacy was 0.91, while the Bartlett's test of sphericity was significant (*p* < 0.001), indicating that the sample was suitable for an EFA.

A two-factor solution fit the data best based on commonly used and recommended criteria, including eigenvalues, scree plot, and parallel analysis (Velicer et al., 2000; Hayton et al., 2004; O'connor, 2012; Yong and Pearce, 2013). The scree plot showed that two factors emerged before the “elbow.” Two factors had an eigenvalue >1, and the results of the parallel analysis demonstrated that two eigenvalues were greater than the comparison eigenvalues (using both the mean and 95th percentile criteria) generated by the parallel analysis, indicating that the two factors should be retained (Hayton et al., 2004). Finally, the two factors explained 69.59% of the variance in the PAWS construct, which met the 60% minimum recommended value (Hinkin, 1998).

Table 1 shows the factor loadings for each item, clustered on their respective factors, represent the correlation of each item with the corresponding factor, While loadings above 0.4 are used commonly to consider a variable as significant (Comrey and Lee, 1992), high factor loading suggest that the measured variable is a good representation of the factor. In this study, all factor loadings were above 0.70 (with a range of 0.74–0.90). The two factors accounted for 69.59% of variance, including the eight items of “Soldiering” (explaining 56.38% of the variance in procrastination at work) and the four items for “Cyberslacking” (explaining 13.22% of the variance). The PAWS and both subscales had good internal consistency coefficients, with 0.93 (total), 0.93 (Soldiering), and 0.87 (Cyberslacking). Correlation tests between the subscales showed that the two factors were significantly correlated with each other (*r* = 0.54, *p* < 0.001).

**Table 1:** Results of the exploratory factor analysis on the PAWS in Sample A.

**Items**	**Factor loading**	
	**F1**	**F2**
**Soldiering**		
PAW 1	**0.85**	
PAW 2	**0.83**	
PAW 3	**0.82**	
PAW 4	**0.80**	
PAW 5	**0.89**	
PAW 6	**0.77**	
PAW 7	**0.74**	
PAW 8	**0.79**	
**Cyberslacking**		
PAW 9		**0.82**
PAW 10		**0.90**
PAW 11		**0.85**
PAW 12		**0.77**
Eigenvalues	6.77	1.59
% of variance explained	56.38%	13.22%

*The bold values are factor loading values of factor 1 and factor 2*.

### Confirmatory Factor Analysis (Sample B)

Sample B revealed medium item communalities (ranging from 0.46 to 0.84) and high factor over-determination (i.e., more than three indicators per factor and two factors in total); therefore, the current sample size (227 > 200) was adequate for factor analysis (MacCallum et al., 1999). Thus, we conducted a series of CFAs for sample B. Four models were tested and compared. In the first model, the two latent PAWS factors were correlated with each other. In the second model, all items of the PAWS loaded on a single latent factor (a unidimensional one-factor model). In the third model, the higher-order model regressed the two factors onto a higher factor. The third model included not only a correlated two-factor model but also a higher-order PAWS factor labeled as PAW. In the last model, the bifactor model had a general PAW factor which allowed 12 items to be freely loaded, along with two uncorrelated factors. The goodness-of-fit indices for these models are reported in Table 2. Table 2 shows that the bifactor model solution for the PAWS has a better fit than the two-factor model, the single factor model, or the second-order two factor model. While the bifactor model had the lowest AIC, this model also generated acceptable fit statistics.

**Table 2:** Confirmatory factor analyses in Sample B.

**Model**	***χ^2^***	***df***	***p***	***χ^2^ /df***	**CFI**	**TLI**	**RMSEA**	**SRMR**	**AIC**	**Comparison**	***χ*** **^2^ Diff test**
1	402.42	54	<0.001	7.45	0.76	0.71	0.17	0.10	6756.83	–	
2	183.29	53	<0.001	3.46	0.91	0.89	0.10	0.06	6539.69	2 vs. 1	219.13 (<0.001)
3	183.29	52	<0.001	3.52	0.91	0.89	0.11	0.06	6541.69	3 vs. 2	1 (1)
4	99.57	42	<0.001	2.37	0.96	0.94	0.078	0.04	6477.98	4 vs. 3	83.72(<0.001)

*Model 1, Single Factor Model; model 2, Correlated Two-Factor Model; model 3, Second Order Two-Factor Model; model 4, Bifactor Model; N = 227; χ^2^ = chi-square statistic; CFI, comparative fit index; TLI, Tucker-Lewis index; RMSEA, root mean square error of approximation; SRMR, standardized root mean square residual; χ^2^ Diff Test, Chi-Squared Difference Test, ANOVA test*.

#### Single Factor Model

This model had poor fit for the data, with [${\text{χ}}_{\left(54\right)}^{2}$ = 402.42, *p* < 0.001], χ^2^/^df^ = 7.45, CFI = 0.76, TLI = 0.71, RMSEA = 0.17, 90% CI [0.15, 0.18], and SRMR = 0.10.

#### Correlated Two-Factor Model

The fit criteria suggested that this model was not a good fit for the data, with ${\text{χ}}_{\left(53\right)}^{2}$ = 183.29, *p* < 0.001, χ^2^/^df^ = 3.46, CFI = 0.91, TLI = 0.89, RMSEA = 0.10, 90% CI [0.09, 0.12], and SRMR = 0.06. All items significantly loaded on factors. Compared with the single factor model, the changes of CFI and RMSEA values were >0.01 (ΔCFI = −0.15, ΔRMSEA = −0.07). We found that the correlated two-factor model demonstrated a better fit than the single factor model, as indicated by a significant chi-squared difference ($\Delta {\text{χ}}_{\left(1\right)}^{2}$ = 219.13, *p* < 0.001).

#### Second-Order Two-Factor Model

This model was not a good fit for the data, with [${\text{χ}}_{\left(52\right)}^{2}$ = 183.29, *p* < 0.001], χ^2^/^df^ = 3.52, CFI = 0.91, TLI = 0.89, RMSEA = 0.11, 90% CI [0.09, 0.12], and SRMR = 0.06. Compared with the correlated two-factor model, the change was very small, and we find that the models were not practically different, as indicated by a chi-squared difference [$\Delta {\text{χ}}_{\left(1\right)}^{2}$ = −2.1404e−09, *p* = 1].

#### Bifactor Model

**Figure 1:**
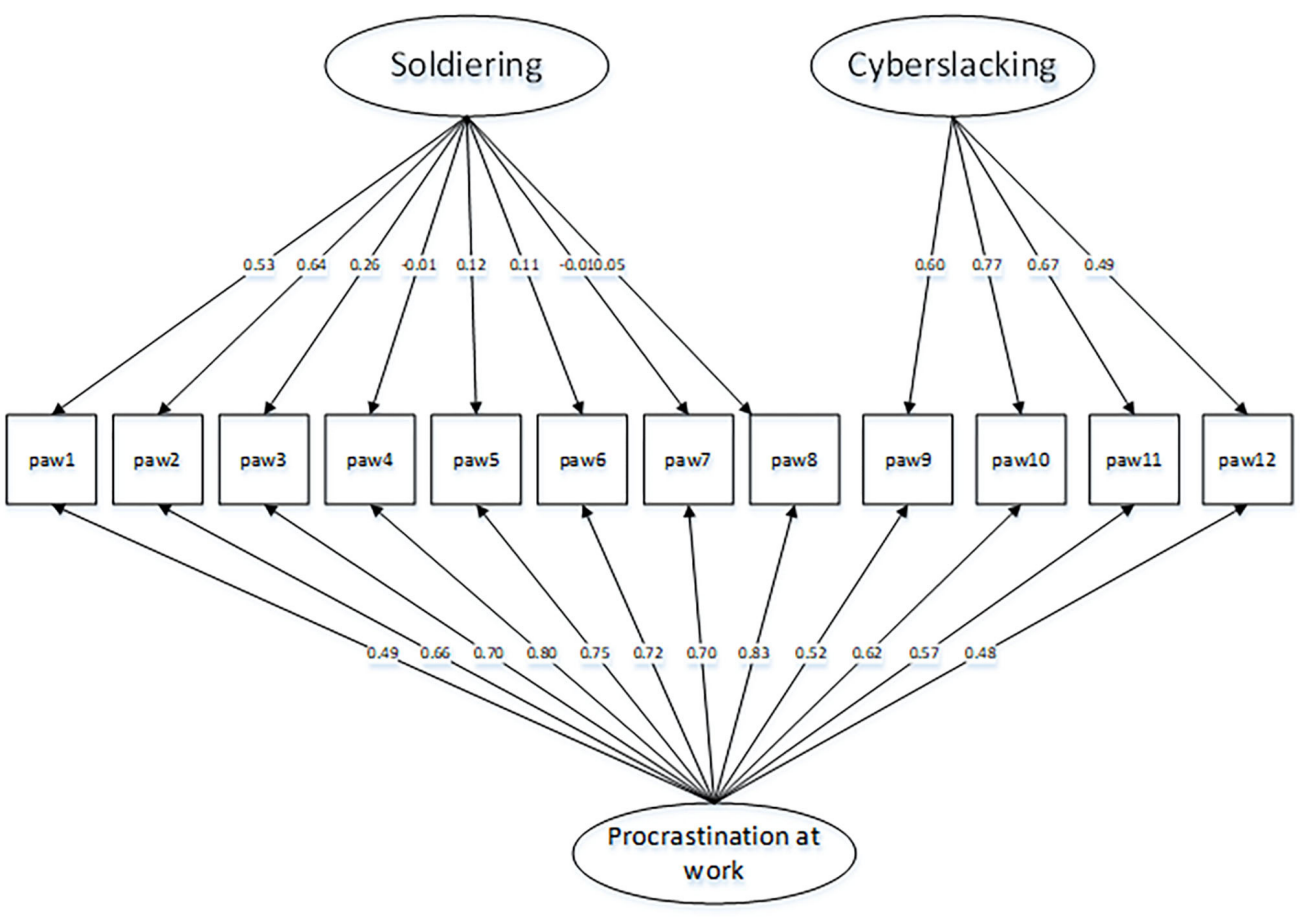
Final confirmatory bifactor model of procrastination at work scale in Sample B. Fit is [${\text{χ}}_{\left(42\right)}^{2}$ = 99.57, *p* < 0.001]; CFI, Comparative Fit Index = 0.96; TLI, Tucker-Lewis Index = 0.98; RMSEA, Root Mean Square Error of Approximation = 0.078; SRMR, Standardized Root-Mean-Residual = 0.04.

This model had a better fit than the correlational model, with ${\text{χ}}_{\left(42\right)}^{2}$ = 99.57, *p* < 0.001, χ^2^/^df^ = 2.37, CFI = 0.96, TLI = 0.94, RMSEA = 0.078, 90% CI [0.06, 0.10], and SRMR = 0.04. Compared with the correlated two-factor model, the change of CFI and RMSEA values were >0.01 (ΔCFI = 0.02, ΔRMSEA = 0.022). We found that the bifactor model demonstrated a better fit than the correlational model, as indicated by a significant chi-squared difference [$\Delta {\text{χ}}_{\left(11\right)}^{2}$ = 83.72, *p* < 0.001]. Therefore, we retained this model as the best fit. Figure 1 depicts this model.

To help interpret the bifactor model and compute model-based statistics, we used the Bifactor Indices Calculator (Dueber, 2017) to calculate relevant bifactor reliability and other coefficients (see Table 3), as recommended by Rodriguez et al. (2015a). In the bifactor model, omega is a reliability estimate for factor analysis that presents the proportion of variance in the PAW total score attributable to common variance rather than error. The omega for the total score, which included the general factors and subscales factors, was 0.94, meaning that 94% of the variance in the total score was due to the factors and 6% was attributable to error. The omegas for the subscales were 0.92 for Soldiering and 0.92 for cyberslacking, indicating high reliability for the general factor and for the specific factors. Omega hierarchical coefficients (ωH) are the proportion of the variance that the general factor contributed to the PAWS total score, coefficient omega hierarchical subscale (ωHS) are the percent of subscale score variance attributable to a group factor (Reise et al., 2013b). When ωH is high (>0.80), ωHS values are low for the two subscales, especially when compared to their corresponding omega values, the majority of reliable variance in subscale scores was attributable to the general factor, a multidimensional construct is considered better at the total score than at the subscale level (Rodriguez et al., 2015b). omegaH for the PAWS total was 0.82, comparing omega (0.94) and omegaH(0.82), that 87.23% of the reliable variance in the PAWS total score (0.82/0.94 = 0.87) is attributable to the general factor, and 14.29% (0.12/0.94 = 0.14) is attributable to the subscale factors. The ωHS was 0.07 (Soldiering) and 0.52 (Cyberslacking), indicating that the reliability of subscale factors decreased owing to the general factor and that the multidimensional PAW construct was more reliable at the total score than at the subscale level.

**Table 3:** Standardized Factor Loading of CFA, IECV, ECV, and model-based reliability estimates according to a bifactor model in sample B.

		**Standardized factor loading**					
**Items**	**IECV**	**General**	**SE**	**Factor 1**	**SE**	**Factor 2**	**SE**
**Soldering**							
Item 1	0.47	0.49	0.08	0.53	0.09		
Item 2	0.51	0.66	0.09	0.64	0.11		
Item 3	**0.88**	0.70	0.07	0.26	0.10		
Item 4	**1.00**	0.80	0.06	−0.01	0.10		
Item 5	**0.98**	0.75	0.07	0.12	0.10		
Item 6	**0.98**	0.72	0.06	0.11	0.10		
Item 7	**0.98**	0.70	0.06	−0.10	0.10		
Item 8	**1.00**	0.83	0.06	0.05	0.10		
**Cyberslacking**							
Item 9	0.42	0.52	0.07			0.60	0.06
Item 10	0.39	0.62	0.07			0.77	0.07
Item 11	0.42	0.57	0.07			0.67	0.07
Item 12	0.49	0.48	0.07			0.49	0.06
ECV		0.68		0.16		0.58	
Ω		0.94		0.92		0.91	
ωH/ωHS		0.82		0.07		0.52	

*Boldface, items with relatively higher IECV (IE., > 0.80); ECV, Explained common variance; Ω, omega; ωH, Omega Hierarchical; ωHS, Omega Hierarchical subscales; IECV, item explained common variance*.

Even if the Ω, ωH, ωHS indicate that the PAWS was more reliable at the general factor level. We also need test whether multidimensional (bifactor) data are “unidimensional enough” to specify a unidimensional measurement model. Explained common variance (ECV) is the proportion of the variance that the general factor accounts for the common variance with all factors (Stucky and Edelen, 2015). The ECV was 0.68 for the PAWS factor and 0.16 and 0.58 for the subscale factors, indicating that the general factor contributed to 68% of the common variance, which explained a greater proportion of common variance than the specific factors. Item explained common variance (IECV), the contribution of each item to the general factor, items with high IECV are good candidates for inclusion on a measure if the goal is to create a unidimensional (one common factor) item set. In the PAWS, six items were relatively high (i.e., six Soldiering items > 0.80), suggesting that Soldiering items mainly explained the variance in the PAWS (Stucky et al., 2013).

The Percent of Uncontaminated Correlations (PUC) represents the percentage of covariance terms that reflect variance only from the general dimension. Along with ECV, PUC influences the parameter bias of the unidimensional solution. According to Reise et al. (2013a), when PUC values are lower than 0.80, general ECV values >0.60 and ωH higher than 0.70 (of the general factor) can also be considered as benchmarks. PUC in our bifactor model was 0.49, ECV was 0.68, and ωH was 0.82, supporting interpretation at the general factor level (Reise et al., 2013a). Thus, we concluded that this scale is best applied at the general or total score level.

### Factorial Invariance (Sample B)

Since the bifactor model had the best fit to the data, we conducted invariance tests to investigate equivalence across gender, age, education level, and job position.

Table 4 shows that the configural model (M0) had a modestly good fit to the data across gender, age, education level, and job position. Indices for gender groups were [${\text{χ}}_{\left(84\right)}^{2}$ = 172.86, *p* < 0.001], CFI = 0.942, RMSEA = 0.097, 90% CI [0.078, 0.117]. Indices for age groups were [${\text{χ}}_{\left(84\right)}^{2}$ = 158.42, *p* < 0.001], CFI = 0.948, RMSEA = 0.087, 90% CI [0.066, 0.108]. Indices for education level were [${\text{χ}}_{\left(84\right)}^{2}$ =191.76, *p* < 0.001], CFI = 0.929, RMSEA = 0.106, 90% CI [0.086, 0.126]. Indices for job position were [${\text{χ}}_{\left(84\right)}^{2}$ =158.26, *p* < 0.001], CFI = 0.952, RMSEA = 0.088, 90% CI [0.067, 0.109]. Fit indices of models all suggested equivalent factor structure across groups and provided a baseline model to compare subsequent models. Next, we tested metric invariance (M1) by constraining all factor loadings to be the same. Changes in fit indicated that the metric model and the configural model did not significantly differ (i.e., ΔCFI ≤ 0.010 and ΔRMSEA ≤ 0.015, Cheung and Rensvold, 2002; Chen, 2007; Rutkowski and Svetina, 2013). Finally, a stronger test of invariance (scalar invariance, M2) was conducted by constraining the item intercepts to be the same across groups. Changes in fit indicated that the metric model and the scalar model did not significantly differ (i.e., ΔCFI ≤ 0.010 and ΔRMSEA ≤ 0.015, Cheung and Rensvold, 2002; Chen, 2007; Rutkowski and Svetina, 2013). Therefore, the factor structure, factor loadings, and indicator intercepts of PAWS were equivalent across groups by gender, age, education level, and job position.

**Table 4:** Test of measurement invariance of the bifactor model across gender, age, education, and job position in Sample B.

**Model**	**χ^2^**	***df***	**CFI**	**RMSEA [90%CI]**	**ΔCFI**	**ΔRMSEA**
**Gender**						
M0 (configural)	172.86	84	0.942	0.097 [0.076, 0.117]		
M1 (metric)	194.70	105	0.942	0.087 [0.068, 0.106]	0.001	0.010
M2 (scalar)	212.03	114	0.936	0.087 [0.069, 0.105]	0.005	0.000
**Age**						
M0 (configural)	156.42	84	0.948	0.087 [0.066, 0.108]		
M1 (metric)	180.84	105	0.946	0.080 [0.060, 0.099]	0.002	0.007
M2 (scalar)	192.13	114	0.944	0.078 [0.058, 0.096]	0.002	0.002
**Edu**						
M0 (configural)	191.76	84	0.929	0.106 [0.086, 0.126]		
M1 (metric)	220.50	105	0.924	0.098 [0.080, 0.117]	0.005	0.008
M2 (scalar)	224.31	114	0.927	0.092 [0.074, 0.110]	0.003	0.006
**Job position**						
M0 (configural)	158.26	84	0.952	0.088 [0.067, 0.109]		
M1 (metric)	186.50	105	0.947	0.083 [0.063, 0.102]	0.005	0.006
M2 (scalar)	199.47	114	0.944	0.081 [0.062, 0.100]	0.003	0.001

*χ^2^, chi-square statistic; CFI, comparative fit index; RMSEA, root mean square error of approximation*.

### Validity Estimates (Sample B)

To assess convergent validity, a measure should be related to theoretically similar constructs (Campbell and Fiske, 1959). As such, the PAWS should relate to, but be distinct from, measures of work engagement and counterproductive work behavior. Bivariate correlations between the PAWS and these variables provide evidence for convergent validity. The correlations between the PAWS and work engagement was −0.23, and the correlation between the PAWS and counterproductive work behavior was 0.44. Results also showed that the AVE for the PAWS (0.59) was higher than 0.50 cut-off, and the average loadings by factor (Soldiering, 0.73; Cyberslacking, 0.84) were all higher than the 0.70 threshold (Hair et al., 2009), which means, on average, 59% of the variations in procrastination at work is explained by these 12 items or questions. In terms of discriminant validity, the correlation analysis conducted between the PAWS and several outcome variables (i.e., task performance, workplace well-being, and organizational commitment) revealed that the AVE for PAWS was higher than the variance that PAWS shared with the outcome variables; the square root of the AVE for the PAWS exceeded the inter-correlations of the PAWS with task performance, workplace well-being, and organizational commitment, showing suitable discriminant validity (see Table 5; Fornell and Larcker, 1981). Finally, as expected, the PAWS showed high significant correlations with constructs that had been shown to have strong correlation in previous studies, thus helping support the nomological validity of the PAWS-C (see Table 5). The results confirmed the convergent, discriminant, and nomological validity of the PAWS.

**Table 5:** Descriptive statistics and correlations in Sample B.

		**Mean**	**SD**	**1**	**2**	**3**	**4**	**5**	**6**
1	PAWS	2.47	0.72	**0.77**					
2	Work engagement	3.52	0.71	−0.23**	**0.85**				
3	Counterproductive work behavior	2.35	0.63	0.44**	−0.29**	**0.62**			
4	Task performance	3.69	0.62	−0.14*	0.46**	−0.16*	**0.73**		
5	Workplace well-being	3.32	0.78	−0.23**	0.59**	−0.26**	0.27**	**0.75**	
6	Organizational commitment	3.22	0.74	−0.29**	0.56**	−0.42**	0.19**	0.66**	**0.69**

(1) * *p < 0.05*,

** *p < 0.01*,

***p < 0.001; (2) In the diagonal (in bold) is the √AVE, the square root of Average Variance Extracted. For PAWS discriminant validity, its √AVE must exceed the inter-correlations of PAWS with all the other constructs (Fornell and Larcker, 1981). Off-diagonal elements below the diagonal are correlations among the constructs.

## Discussion

The 12-item PAWS is a recently developed measure to assess procrastination in the work context. This study aimed to test the scale structure and validity of the Chinese version of the PAWS, and it is the first to employ second-order factor CFA and bifactor CFA to examine the validity of the PAWS total score. It is also the first study to examine the factorial invariance of the PAWS across groups by gender, age, education, and job position among Chinese employees. Our study contributes to the literature on procrastination at work by providing further validation evidence for PAWS, as previous studies have not fully examined counterproductive behavior in the work context (van Eerde, 2003; Metin et al., 2016).

The EFA conducted in stage 1 indicated that the PAWS-C consisted of two factors that represented distinct subscales, the results provided preliminary support for a multidimensional measure of procrastination at work with reliable response, consistent with the original study (Metin et al., 2016). Results with excellent internal consistency coefficients suggest that the 12-item PAWS accurately assesses two domains of procrastination at work, soldiering and cyberslacking. To confirm the factor structure of the PAWS-C obtained from stage 1, CFA was conducted. We examined four models in stage 2: a single factor model, a correlated two-factor model, a second order two-factor model, and a bifactor model. The bifactor model was the best fit to the data, indicated that the PAWS-C items share a common, underlying factor while also loading onto their own sub-factors. Results suggested that majority of the reliable variance in the PAWS-C total score is attributable to the general factor, meantime, more than two-thirds (68%) of the common variance was attributable to the general factor, which suggests a stronger general factor. Therefore, using a manifest total score is warranted, in the future, researchers should ideally represent procrastination at work as a bifactor model within a latent framework, this type of analysis is best for separating the relative common variance associated with the general factor and the subscales, respectively. Both EFA and CFA results supported a two-factor of PAWS-C in sample A and sample B. Result of bifactor model test indicated that the most meaningful interpretation of the PAWS is achieved at the general factor or total score level. Based on these results, we recommend that the PAWS be applied at the general or total score level, instead of using subscales alone to measure Soldiering or Cyberslacking in the work context. As mentioned above, Cyberslacking is harder to measure than Soldiering, EFA results provide evidence that lower explained variance (only 13.22% in EFA) was found in Cyberslacking, however, Cyberslacking subscale also is important for PAWS, bifactor CFA results showed higher ECV, ωH compared to Soldiering in CFA, the four items Cyberslacking subscale is reliable and meaningful.

The results also showed that the PAWS was related to, but distinct from, measures of work engagement and counterproductive work behavior. Nomological validity was supported by finding expected positive/negative correlations with other closely related concepts and other constructs within the nomological network. Results showed a strong relationship between PAW and counterproductive work behavior. This is consistent with previous studies: “procrastination at work as a counterproductive behavior” and “counterproductive work behavior” are independent yet related concepts (Metin et al., 2016). Moreover, PAW was associated with low work engagement, low task performance, low workplace well-being, and low organizational commitment. Employees with high levels of PAW may be less likely to engage in work activities because they spend more time on non-work activities (Metin et al., 2018), which also means low work output (Steel, 2007). Procrastination as a counterproductive behavior negatively affects the well-being of employees, and employees with high levels of PAW also have low levels of organizational commitment (Wertenbroch, 2002) and are more likely to leave the organization. Comparing with Gupta et al. (2012) study, the present study conceptualized procrastination as work behavior, results show procrastination at work include soldiering and cyberslacking. In Gupta et al. (2012) study, they conceptualized procrastination as a stable and enduring personality trait, and used general procrastination scale to measure procrastination, examine the extent to which five qualitatively different types of time perspective predict the tendency to procrastinate in the workplace. In the future, we could examine whether time perspective as a personality trait can predict the procrastination behavior at work using PAWS-C.

Additionally, we tested measurement invariance across gender, age, education, and job position. Three models—configural, metric, and scalar—were run and fit did not substantially decline for any of these models across any of the groupings. This suggests that the general structure of the scale and responses to the scale itself may be equally valid between participants within these groups. This means that people may have different levels of procrastination at work based on their demographics, but PAWS measures procrastination at work in the same way.

Understanding and improving procrastination at work is important to both employees and organizations. In practice, the present results offer a tool and do give some support for using this measure in the Chinese context. Managers and employees might find it useful for themselves to assess the extent to procrastination at work. The results of the present study suggest that two components of procrastination at work is unique. Managers can use PAWS to measure and know the degree of their subordinate's procrastination at work that can help managers most effectively target specific parts of employee's work design and task Progress that need follow up and improvement (Prem et al., 2018). Employees using this assessment tool may help to manage their work time and make a work plan better. For example, for employees who Cyberslacking, they can try turning off their cell phones during work hours or disconnecting their computers from the Internet.

This study had several limitations. Its small sample size calls for future research to replicate these results among larger populations to allow for generalizability. Because most our sample reported PAWS as about average, we were unable to establish cut-off scores to differentiate between low, medium, and high levels of procrastination at work. We were also unable to test measurement invariance across different PAW level groups, which future research should examine (Meredith, 1993). This study validated results only among Chinese employees; therefore, researchers should use caution when expending the present results to other languages in China and other Mandarin-speaking cultures outside China. To expand on the present results, future studies should also assess scale properties in other languages and cultures. In this study, we did not use general procrastination scales such as General Procrastination Scale (Lay, 1986), the Irrational Procrastination Scale (Steel, 2002) to test the construct validity, the main consideration is that these scales have not been validated in the Chinese context. In the future, we should consider validate these scales in the Chinese context and comparing them with the PAWS-C. Should be mentioned, although Aitken's Procrastination Inventory (Aitken, 1982) was validated in the Chinese context, but the sample was students, Research showed that procrastination was negatively correlated with age (Gupta et al., 2012), and the author did not test for measurement invariance (Chen and Dai, 2008).

## Conclusion

Our results show that the PAWS is reliable and valid in Mandarin and for the Chinese culture and can be used for research on procrastination in Chinese employees. This study found that a bifactor model best fit the underlying structure of the Chinese version of PAWS, and that the use of the PAWS-C total score is valid. In addition, the bifactor structure of PAWS-C is equivalent across gender, age, education, and job position groups. The PAWS-C was related to counterproductive work behavior and work engagement and was also significantly correlated with other constructs in the nomological net.

## Data Availability Statement

The original contributions presented in the study are included in the article/Supplementary Material, further inquiries can be directed to the corresponding author/s.

## Author Contributions

All authors have made substantial intellectual contribution to the study, and approved it for publication.

## Funding

National Science Foundation of China (Grant No. 71772171) and Public Computing Cloud, Renmin University of China.

## Conflict of Interest

The authors declare that the research was conducted in the absence of any commercial or financial relationships that could be construed as a potential conflict of interest.

## Publisher's Note

All claims expressed in this article are solely those of the authors and do not necessarily represent those of their affiliated organizations, or those of the publisher, the editors and the reviewers. Any product that may be evaluated in this article, or claim that may be made by its manufacturer, is not guaranteed or endorsed by the publisher.
