# Supplementary material for: Validation of the Chinese Version of the Procrastination at Work Scale
Source: Front Psychol. 2021 Sep 16;12:726595. doi: 10.3389/fpsyg.2021.726595 (PMC8481865; doi:10.3389/fpsyg.2021.726595)
Supplement: Supplementary file 1 [file Table_1.DOCX]

**Supplementary material**

**Table 1|** **Demographic information about the participants**

| **Sample A (n=236)** | | |
| --- | --- | --- |
|  | **Mean** | **Standard deviation** |
| **Age** | 34.29 | 8.22 |
| **Tenure** | 11.97 | 8.98 |
| **Working hours per week** | 47.85 | 13.49 |
| **Gender** | **Frequency** | **Percentage** |
| Male | 132 | 55.93% |
| Female | 104 | 44.07% |
| **Education** | **Frequency** | **Percentage** |
| bachelor’s degree or below | 151 | 63.98% |
| master’s degree or above | 85 | 36.02% |
| **Position** | **Frequency** | **Percentage** |
| Manager (first-line/middle/senior) | 142 | 60.17% |
| Front-line employee | 94 | 39.83% |
| **Sample B (n=227)** | | |
|  | **Mean** | **SD** |
| **Age** | 34.68 | 7.77 |
| **Tenure** | 11.91 | 8.85 |
| **Working hours per week** | 48.09 | 11.24 |
| **Gender** | **Frequency** | **Percentage** |
| Male | 125 | 55.07% |
| Female | 102 | 44.93% |
| **Education** | **Frequency** | **Percentage** |
| bachelor’s degree or below | 143 | 63.44% |
| master’s degree or above | 83 | 36.56% |
| **Position** | **Frequency** | **Percentage** |
| Manager (first-line/middle/senior) | 136 | 59.91% |
| Front-line employee | 91 | 40.09% |

**Table 2| Procrastination at Work Scale**

| **Soldiering (磨洋工)** |
| --- |
| Q1. When I work, even after I make decision, I delay acting upon it.  在工作中，即使做了计划，我也会延迟执行。 |
| Q2. I delay before starting on work I have to do.  在开始必须要做的工作之前，我会拖延。 |
| Q3. At work, I crave a pleasurable diversion so sharply that I find it increasingly hard to stay on track.  在工作时，我会因为非常渴望休闲放松，而越来越难进入工作状态。 |
| Q4. When a work task is tedious, again and again I find myself pleasantly daydreaming rather than focusing.  当工作任务无聊时，我会发呆走神，难以专注工作。 |
| Q5. I give priority to the lesser tasks, even if there is something important, I should do at work.  尽管有更重要的事情要做，我也会先处理轻松的任务。 |
| Q6. When I have excessive amount of work to do, I avoid planning my tasks, and find myself doing something totally irrelevant.  当工作任务繁重时，我会逃避制定计划，并去做一些与工作无关的事情。 |
| Q7. I take long coffee breaks.  工作中休息时，我会尽量休息久一点。 |
| Q8. I delay some of my tasks just because I do not enjoy doing them.  对一些不喜欢的工作，我会拖延。 |
| **Cyberslacking (网络摸鱼)** |
| Q9. I use Instant Messaging (i.e., WhatsApp, Skype, Viber. . .) at work for personal use.  上班时间，我因为私事使用聊天软件（如微信、QQ等）。 |
| Q10. I spend more than half an hour on social network sites (Facebook, Instagram, Twitter etc.) at work per day for leisure purpose.  上班时间，我花在社交网站（如微博、知乎、朋友圈等）的时间超过半小时。 |
| Q11. I read news online at work.  上班时间，我上网看新闻。 |
| Q12. I do online shopping during working hours.  上班时间，我网上购物。 |
